# Study protocol: a randomized controlled trial of a computer-based depression and substance abuse intervention for people attending residential substance abuse treatment

**DOI:** 10.1186/1471-2458-12-113

**Published:** 2012-02-10

**Authors:** Peter J Kelly, Frances J Kay-Lambkin, Amanda L Baker, Frank P Deane, Adam C Brooks, Alexandra Mitchell, Sarah Marshall, Meredith Whittington, Genevieve A Dingle

**Affiliations:** 1 Illawarra Institute for Mental Health, School of Psychology, University of Wollongong, Northfields Avenue, Wollongong 2522, Australia; 2 National Drug and Alcohol Research Centre, University of New South Wales, King Steet, Sydney 2052, Australia; 3 Centre for Brain and Mental Health Research, University Drive University of Newcastle, Callaghan 2308, Australia; 4 Treatment Research Institute, Independence Mall West, Philadelphia 19106, USA; 5 School of Psychology, University of Queensland, Sir Fred Schonell Drive, Brisbane 4072, Australia

## Abstract

**Background:**

A large proportion of people attending residential alcohol and other substance abuse treatment have a co-occurring mental illness. Empirical evidence suggests that it is important to treat both the substance abuse problem and co-occurring mental illness concurrently and in an integrated fashion. However, the majority of residential alcohol and other substance abuse services do not address mental illness in a systematic way. It is likely that computer delivered interventions could improve the ability of substance abuse services to address co-occurring mental illness. This protocol describes a study in which we will assess the effectiveness of adding a computer delivered depression and substance abuse intervention for people who are attending residential alcohol and other substance abuse treatment.

**Methods/Design:**

Participants will be recruited from residential rehabilitation programs operated by the Australian Salvation Army. All participants who satisfy the diagnostic criteria for an alcohol or other substance dependence disorder will be asked to participate in the study. After completion of a baseline assessment, participants will be randomly assigned to either a computer delivered substance abuse and depression intervention (treatment condition) or to a computer-delivered typing tutorial (active control condition). All participants will continue to complete The Salvation Army residential program, a predominantly 12-step based treatment facility. Randomisation will be stratified by gender (Male, Female), length of time the participant has been in the program at the commencement of the study (4 weeks or less, 4 weeks or more), and use of anti-depressant medication (currently prescribed medication, not prescribed medication). Participants in both conditions will complete computer sessions twice per week, over a five-week period. Research staff blind to treatment allocation will complete the assessments at baseline, and then 3, 6, 9, and 12 months post intervention. Participants will also complete weekly self-report measures during the treatment period.

**Discussion:**

This study will provide comprehensive data on the effect of introducing a computer delivered, cognitive behavioral therapy based co-morbidity treatment program within a residential substance abuse setting. If shown to be effective, this intervention can be disseminated within other residential substance abuse programs.

**Trial registration:**

Australia and New Zealand Clinical Trials Register (ANZCTR): ACTRN12611000618954

## Background

It is very common for people with alcohol or substance dependence disorders to also have other co-occurring mental illnesses. This is particularly the case for people attending residential alcohol and other substance abuse treatment services, where it is estimated that between 64% and 71% of participants have co-occurring mental illness [1]. When compared to people with a substance abuse problem unaccompanied by mental illness, people with co-occurring mental illnesses tend to have much poorer treatment outcomes. For example, they are significantly more likely to have a poor treatment response, higher rate of relapse, more hospital visits, increased incidence of Hepatitis C and HIV infection, higher incarceration rate, family difficulties and homelessness [see 2].

Traditionally, alcohol and other substance abuse services have delivered drug and alcohol interventions and relied on mental health services, working in either a parallel or sequential fashion, to address the mental health of their patients [3,4]. This type of approach has proven largely ineffective, often resulting in increased fragmentation between the services, higher treatment dropout and exclusion of participants from substance abuse services [5]. In response, integrated approaches have been developed that concurrently target both the substance abuse and mental illness [see 3 review]. There is evidence that integrated residential treatment facilities are more effective in treating complex mental illness and substance abuse disorders, than less integrated facilities [2].

Cognitive Behavioural Therapy (CBT) is an approach that lends its self very successfully to being delivered in an integrated fashion. There is strong support for its effectiveness in the treatment of alcohol and substance abuse disorders [6], as well as across a wide range of psychiatric conditions [7]. Whilst CBT has demonstrated comparative effectiveness to other therapeutic approaches [8], it tends to have more durable long-term effects when compared to other treatment conditions [9]. For example, in a randomized trial comparing integrated CBT with a12-Step facilitation therapy for people diagnosed with substance dependence and depression, both interventions produced similar reductions in substance use and depression at the end of treatment [10]. However, participants in the integrated CBT condition continued to demonstrate improvements in their depression at 6-months follow-up, while depression for participants in the 12-Step condition deteriorated. More stable reductions in substance use were also demonstrated for people in the integrated CBT condition at follow-up.

Whilst there is increasing empirical support for integrated treatment approaches, the majority of substance abuse services do not address co-occurring mental illness in a systematic way. For example, a review of residential substance abuse services across three Australian states indicated that 88% of services were not equipped to provide integrated mental health treatment [11]. This appears to be largely the result of organisations employing people with limited or no mental health qualifications and failing to prioritise co-morbidity treatment [12]. Moreover, high staff turnover rates in the substance abuse field [13,14] makes attempts to train staff in new therapeutic approaches, such as integrated CBT, problematic and difficult to sustain in the longer term.

One approach to improve the utilization of evidence-based approaches in health settings is to use computer based interventions [15]. Benefits of computer based approaches are that they do not require additional staff, do not rely on staff having specialist mental health or substance abuse training, and are relatively cost-efficient to deliver. Emerging research has demonstrated that computer delivered interventions can produce clinically significant improvements across a range of outcome domains [16]. Computer delivered interventions are increasingly being recommended for use in the substance abuse field [17-21]. Whilst it is still a developing area of clinical research, the use of computer based CBT interventions for drug and alcohol abuse appears to be efficacious [22]. For example, CBT4CBT is a 6-session, computer-delivered program that is based on cognitive behavioural principles. It is a self-directed program that is used as an adjunct to treatment as usual. Evaluation of the program indicated that it is superior to treatment as usual, both at the end of treatment, and at 8-weeks and 6-month follow-up [23,24]. Whilst CBT4CBT appears to be quite a promising intervention, it does not specifically target co-occurring mental illnesses.

An integrated computer delivered CBT intervention for co-occurring depression and alcohol or substance use disorders has been developed. The Self-Help for Alcohol/other drug use and Depression program [SHADE; 25] is a 10-session multimedia psychotherapy treatment program incorporating motivational, behavioural and cognitive components. The SHADE program delivers its therapeutic content via a number of interactive components including video demonstrations, voiceovers, and in session exercises. Results from an initial randomised clinical trial indicated that the SHADE Program outcomes were equivalent when a therapist delivered the same intervention to people attending outpatient substance abuse treatment. It also produced significantly better treatment outcomes than a brief intervention alone [25]. To date research has not examined the use of SHADE within a residential treatment setting or the extent that it might enhance standard care [26].

The current project will be conducted at The Salvation Army Sydney Recovery Service Centres located in the Australian states of Sydney and Brisbane. These centres provide a 12-step based residential alcohol and other substance abuse program. The purpose of the current study is to examine the effectiveness of 'adding' the SHADE program to an already established treatment facility. The study will be conducted as a randomized trial, in which participants allocated to the treatment condition will complete the SHADE Program. A typing training program will be used as an active control condition. As mental illness is not specifically targeted as part of The Salvation Army program, and CBT is not systematically available for substance use disorders, it is hypothesized that individuals in the Treatment Condition will report greater improvements in their mental health and greater reductions in their substance use at follow-up, than individuals in the Control Condition. Recruitment for the study is currently underway. The study is funded by a competitive research grant from the Australian Rotary Health Fund. The University of Wollongong Human Research Ethics Committee (HE11/091) has approved the research trial, which is registered with the Australian New Zealand Clinical Trials Registry (ACTRN12611000618954).

## Methods/Design

### Study setting

The research program is being conducted at two sites, the Sydney Recovery Service Centre and the Brisbane Recovery Service Centre. The Australian Salvation Army operates both of these centres. The treatment program is up to 10-months in length and is operated in the form of a modified therapeutic community. The Sydney service has 102 beds, including 82 for males and 20 for females. The Brisbane service has 84 beds, including 72 for males and 12 for females. All participants are required to be over the age of 18 and must have an alcohol, substance abuse or gambling problem.

### Study design

**Figure 1:**
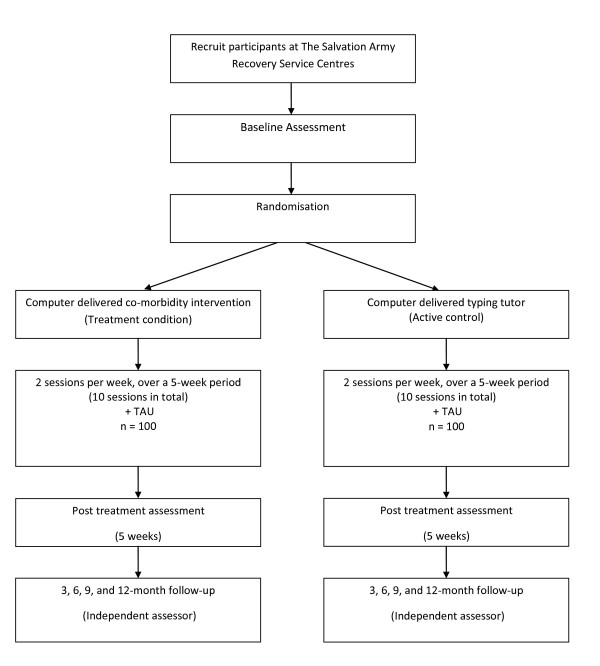
**Study design**.

This is a prospective randomised controlled trial. Figure 1 shows the design of the study. After completion of a baseline assessment, participants will be randomly assigned to either the treatment condition (i.e. computer delivered integrated substance abuse and mental health intervention; SHADE) or an active Control Condition (i.e. computer delivered typing tutorial program; Type Master Pro). Randomization will be stratified by gender (male and female), length of time the person has been in the program (4-weeks or less, 4-weeks or more) and current use of anti-depressant medication (currently taking anti-depressant medication, not taking anti-depressant medication). Following completion of the baseline assessment for each participant, the researchers will be issued with a sealed randomisation envelope by an independent researcher that displays the participant identification code. Participants will be instructed to open the envelope at the conclusion of the initial assessment. A permuted block randomisation approach will be used so that the distribution of participants across treatment conditions will be maintained regardless of the final sample size.

### Participants

All participants will be attending either the Sydney or Brisbane Recovery Service Centres. For the purpose of the current study, only people diagnosed with an alcohol or substance abuse problem will enter the study. It is expected that over the course of the study approximately 200 people will be recruited to participate. Previous surveys of people attending Australian Recovery Service Centres indicate that on average participants are 37 years of age (SD = 9.90) and have had long-term substance abuse problems (M = 19 years; SD = 23.10). On average, alcohol is the most commonly reported primary substance of abuse (55.6%), followed by amphetamines (20.8%), cannabis (9.7%), heroin (6.2%) and others [7.7%; [27]]. All participants will complete informed written consent prior to commencing the study, and all participants will be over the age of 18.

### Inclusion and exclusion criteria

All participants attending the residential program will be approached to participate in the study. Exclusion criteria have been kept to a minimum to ensure that the study can examine the effectiveness of using the SHADE program within a 'real world' setting. Participants will only be excluded from the study if: a) the person did not satisfy the diagnostic criteria of an alcohol or substance dependence disorder (i.e. people who were attending the Recovery Service Centre for only gambling). b) The person is a non-English speaker or has a low level of literacy or an organic brain disease that would impact the persons understanding of the computer interventions.

Participants will be included in the study even if they do not have a current or previous diagnosis of a depressive disorder. This decision was made for four reasons: a) approximately 80% of participants attending The Salvation Army Recovery Service Centres report a lifetime history of symptoms associated with depression [1]. Subsequently, people with alcohol and substance abuse disorders are at risk of developing depression; b) Individuals with substance abuse disorders typically experience high levels of depressotypic thoughts [28]; c) Previous research shows that symptoms of depression at follow up rather than a diagnosis of a mood disorder at intake is associated with relapse following treatment for substance use disorders [29] and; d) it is expected that participants without symptoms associated with depression would still benefit from the substance abuse component of the intervention. To account for the potential confound of depressive disorders participants will be stratified based on their current use of anti-depressant medication as part of the randomization procedures.

### Interventions

#### Treatment Condition (computer based co-morbidity treatment)

Individuals in this condition will complete the SHADE program [25]. Participants will attend two × 1-hour sessions each week, completing the program in five-weeks (approximately 10 h of engagement with SHADE in total). These sessions will be conducted on computers located at the Recovery Service Centre. Research assistants will provide technical support whilst participants are completing the program (e.g. helping the person to become familiar with using the program), but will not provide any clinical support. The SHADE program is a clinical intervention that incorporates aspects of motivational interviewing and CBT. The program content is delivered in an integrated fashion and is designed to encourage a reduction in depression and alcohol and/or substance use. The program contains interactive components, including video demonstrations, voiceovers and in-session exercises. The video components are viewed and model the cognitive and behavioural skills relevant to the therapy (e.g. activity scheduling, self-monitoring of thoughts, challenging faulty cognitions, identifying and evaluating cognitive schema, drink/drug refusal and problem solving).

#### Active Control Condition (computer delivered typing tutor program)

A limitation with previous computer based intervention studies is the lack of an active control condition [22]. To address this concern we have included a computer delivered typing tutor to control for the amount of screen time participants in the treatment condition experience. Participants in the active control condition will complete a commercially available typing tutor program [30]. This program has previously been used as an active control in studies examining computer delivered cognitive remediation for participants attending residential substance abuse services [31]. Participants will attend two × 1-hour sessions each week, completing the computer delivered typing program in 5 weeks (approximately 10 h in total). This is comparable to the Treatment condition. These sessions will be conducted on computers located onsite and, as with the Treatment condition, research staff will be available to support participants with the technical aspects associated with using the computer program.

#### Treatment as usual (TAU)

Participants in both the Treatment and Control conditions will be completing The Salvation Army Recovery Service Centre program (i.e. TAU). The program utilises a 12-Step approach and is primarily based on the disease model of addiction. Participants attend individual case management, group therapy sessions and regular chapel services. Groups provided during the program cover a diverse and eclectic range of areas including social skills training, relapse prevention planning, family systems work and anger management. However, participants do not have access to cognitive behavioural therapy. The program does not specifically address co-occurring mental illness, although participants may be under the care of an external medical practitioner and be taking psychiatric medication.

#### Outcome measures

Baseline assessments will be conducted prior to notification of randomization status. This will include administering the Structured Clinical Interview of the DSM IV [SCID; [32]] to identify 12-month and lifetime history of alcohol or other substance abuse and dependence disorders. Additionally, 12-month and lifetime history of depressive disorders will also be assessed using the SCID. Outcome measures will be collected at baseline, weekly during the 5 weeks of the treatment program and then at 3-, 6-, 9-, and 12-month follow-up (see Table 1 for schedule). Independent assessors, who will remain blind to intervention allocation, will conduct follow-up telephone assessments to collect this data. Each participant will be offered $20 AUD for completion of the baseline assessment and each subsequent follow-up assessment, as reimbursement for the time associated with completing the measures. Participants will receive $10 AUD for each assessment completed during the treatment program. For assessments completed while a participant is a resident at the Recovery Service Centre, he or she will be remunerated in cash. For telephone assessments completed once the participant leaves the program, gift certificates will be mailed out.

**Table 1:** Assessment instruments proposed for the current study

Domain assessment and instrument used	Baseline	Weeks	Week	Months
		
		1 to 4	5	3, 6, 9,12
**Diagnostic Assessment**				

Structured Clinical Interview for DSM Disorders: depression and substance abuse sections only	✓			

**Alcohol and substance abuse measures**				

Addiction Severity Index: Alcohol and Drug composite scores	✓		✓	✓

Alcohol Use Disorders Identification Test	✓			

Drug Abuse Screening Test	✓			

Opiate Treatment Index	✓		✓	✓

Drug Taking Confidence Questionnaire	✓	✓	✓	✓

PENN Alcohol Craving Scale	✓	✓	✓	✓

Drug Risk Response Test	✓		✓	

Fagerstrom Test for Nicotine Dependence	✓	✓	✓	✓

**Mental health measures**				

Addiction Severity Index: Mental Health composite score	✓		✓	✓

Depression, Anxiety and Stress Scale	✓	✓	✓	✓

Beck Fast Screen	✓	✓	✓	✓

Dysfunctional Attitudes Scale	✓	✓	✓	✓

Ways of Responding	✓		✓	

**Other**				

Background and demographic information	✓			

Addiction Severity Index: Employment Status composite score	✓			✓

Client Satisfaction Survey			✓	

Computer Attitudes Scale	✓	✓	✓	

#### Primary outcome measures

The two primary outcome variables will be: a) level of substance use; and b) level of depression. The level of substance use will be measured using the composite scores for alcohol use and drug use from the Addiction Severity Index - 5^th ^Edition [ASI; [33]]. The ASI is a semi-structured clinical interview that has been used widely in addiction research [34]. The Opiate Treatment Index [OTI; [35]] will be used as an alternate measure of alcohol and substance use in the last month. The OTI yields a score reflecting the mean number of substance use occasions per day for each drug class assessed for the month prior to assessment. The Timeline Follow-Back Method [TLFB; [36]] will be used to improve participants' recall of their alcohol and substance use on the ASI and OTI. The TLFB method yields high test-retest reliability and has been used successfully in both face-to-face and telephone delivered assessment procedures [37,38]. The Beck Depression Fast Screen [BDFS; [39]] and the depression subscale of the Depression, Anxiety and Stress Scale [DASS-21; [40]] will be used to examine changes in depression levels during the course of the study.

#### Secondary outcome measures

A requirement of treatment in the residential facility is that participants do not use any alcohol or non-prescribed substances of abuse. Therefore, to be able to examine alcohol and substance use outcomes whilst a person is a resident of the program, secondary outcome measures will be included that examine cravings for the person's primary substance of abuse [PENN Alcohol Cravings Survey; [41]] and self-efficacy in terms of resisting the urge to drink alcohol or take drugs in specific high relapse risk situations [Drug Taking Confidence Questionnaire; [42]]. Additionally, the Dysfunctional Attitudes Scale [43] will be used to examine changes in the existence and intensity of dysfunctional attitudes commonly associated with depression. The ASI composite score for employment status will also be used to examine employment outcomes.

To examine the potential mechanisms of change associated with the SHADE program, two measures of coping skill acquisition will be included. The Drug Risk Response Test [DRRT; [44,45]] measures coping skills to manage circumstances in which there is a high risk for using alcohol or other drugs. It has been used in a number of studies exploring coping skill acquisition amongst individuals receiving treatment for substance use disorders [45-47]. The Ways of Responding Questionnaire [WOR; [48]] is a measure that examines coping skills in response to situations that increase the risk of depressed mood. Both the DRRT and the WOR involve the participant providing responses to hypothetical scenarios, with their responses providing an indication of both their adaptive and maladaptive coping skills. Research assistants blind to the participants treatment condition will rate both the WOR and the DRRT.

### Data-analysis

#### Power analysis

Data from the SHADE study [25] indicate that the computerized intervention is associated with a 1.53 effect size change for depression and 0.86 for alcohol between baseline and 12-month follow-up assessments. These effects sizes were entered into G-Power 3.1 [49] in order to estimate the sample sizes required to detect similar differences between the treatment and active control conditions in the current study. Power was set at 80% and conservative 2-tailed tests were assumed even though a directional hypothesis is proposed. The sample size needed to detect a similar effect size between groups on depression is 8 per group for depression (total N = 16) and 23 per group for alcohol (total N = 46). Given the SHADE studies compared the computerized intervention to a control condition consisting of only received a brief intervention and the proposed study is comparing SHADE + TAU to an active control condition + TAU, we anticipate smaller effect sizes. When a more conservative medium effect size of .50 is used, power is set to 80% and a 2-tailed test is used, a sample size of 64 per group is needed (total N = 128). A conservative drop out rate is 35% of participants at follow-up. As such, we will recruit a minimum of 200 participants to the study. This will ensure that we will have sufficient power to conduct the analysis.

#### Analysis plan

Data coding and analysis will be carried out by the authors using available software packages. Variables hypothesised to change over time according to treatment allocation will be examined predominantly using generalized linear mixed models. These techniques facilitate management of missing data without imputing values or excluding participants. Primary outcome measures will typically be analysed in two ways: a) intention to treat; and b) analyses performed within the sub-sample of participants who completed the majority of treatment sessions. In addition, comparisons on selected demographic and clinical characteristics will be made between this sub-sample and those who dropped out of treatment, to help detect any biases in outcome measures. Other potential confounders will also be examined (e.g. involvement in additional mental health treatments) and their potential effects will be modelled in the major analyses (e.g., controlling and not controlling for these variables). As a partial control for the number of statistical tests to be conducted, the threshold for significance will be set at p < 0.01.

## Discussion

The present study is examining the effectiveness of 'adding' a computer delivered co-morbidity intervention to residential alcohol and other substance abuse treatment. A large proportion of people accessing residential drug and alcohol treatment screen positive for mental illness, with depression being the most common [1]. It is expected that long-term mental health outcomes for participants who complete the SHADE program will be significantly better than for those allocated to the control condition. Additionally, as the residential treatment facility is based on a 12-step approach, rather than a CBT approach, it is expected that the CBT based SHADE intervention will also improve alcohol and substance abuse outcomes for participants relative to the control condition.

### Strengths and limitations

A significant strength of the current research is that it will be conducted as an effectiveness study. Unlike efficacy studies, where clinical trials are typically conducted in highly controlled research environments, effectiveness studies are conducted in 'real world' treatment settings. The advantage of using this approach is that the results are more representative of 'actual' clinical practice and provide evidence regarding the feasibility of using the intervention as part of ongoing routine care. The research design also includes additional attempts to increase the generalizability of the results by using very inclusive eligibility criteria.

Previous trials of the SHADE program have used therapists to support the delivery of the program [25,26]. This has included the delivery of a one-session intervention at the commencement of treatment and 10-minute 'check in' sessions at the conclusion of each computer session. Possible advantage of such an approach are that therapists can check participants understanding of the intervention, confirm homework assignment, and therapists can address any motivational issues. It has been suggested that therapist support is likely to improve client utilisation of computer interventions and subsequently improve client outcomes [18]. However, a decision has been made in this study to not provide therapist support in the delivery of the program. This is in line with previous computer delivered interventions for substance abuse clients [44] and likely reflects more closely how interventions would ultimately be implemented within routine care.

A further strength is that the research design includes an active attention control condition. A computer delivered typing tutor will be used as the control, with participants being provided with the rationale that improving their typing skills will help their future employment opportunities. This active control has previously been used in computer based trials in residential alcohol and other substance abuse treatment settings [31], and addresses limitations with previous clinical trials in which comparisons have only been made to no-treatment conditions [22].

A significant challenge for this project will be participant dropout due to participants leaving the residential treatment facility prior to completing the intervention. Dropout rates from substance abuse treatments are extremely high. Fifty-seven percent of participants prematurely leave within the first 3-months of treatment [50]. Similar percentages have also been reported in the broader alcohol and other substance abuse treatment literature [see [51] for review]. To help address this concern, participants will complete the SHADE or computer delivered typing interventions twice a week (i.e. over a 5-week period). This represents a shift from the protocol used by Kay-Lambkin et al. [25], where SHADE was delivered weekly for 10-weeks.

A further challenge for the study will be retaining participants at follow-up. People with alcohol and other substance abuse disorders are traditionally very difficult to follow-up. This is further complicated with residential facilities, as participants often move outside of their local area to attend treatment. Attempts to improve follow-up rates in the current study will include using telephone follow-up, obtaining contact details of significant others to help with locating participants, reinforcing to participants the importance of conducting follow-up and financially compensating participants for the time required to complete the assessments (AUD$20).

## Conclusion

To our knowledge this is the first study to examine the use of a computer based comorbidity intervention within a residential substance abuse setting. The current study seeks to address a significant gap in treatment delivery by examining the effectiveness of using a computer-based comorbidity intervention, employing CBT strategies, within a residential substance abuse setting. Results from this study will provide very valuable information regarding effectiveness of 'adding' the SHADE program to routine care. The study will also provide information regarding the feasibility of using computer delivered interventions within residential alcohol and other substance abuse settings.

## Competing interests

Kelly and Deane both hold research consultancies with The Salvation Army. The SHADE computer based program has been licensed for use in the United States of America by Cobalt Therapeutics. The authors (FKL, AB) receive no financial benefit as a result of this licensing agreement.

## Authors' contributions

All authors have made an intellectual contribution to this research trial. The study chief investigators PJK, FKL, AB, FPD, ACB and GAD were responsible for identifying the research questions, design of the study and overseeing the implementation of the study. Associate investigator AM was responsible for the development of additional research questions, selection of process measures and development of inter-rater reliability procedures. Research assistants SM and MW contributed to the development of support materials, recruitment of participants and study implementation. All authors were responsible for drafting of this manuscript and have read and approved the final version.

## Pre-publication history

The pre-publication history for this paper can be accessed here:

http://www.biomedcentral.com/1471-2458/12/113/prepub
